# Determination of growth stages and metabolic profiles in *Brachypodium distachyon* for comparison of developmental context with Triticeae crops

**DOI:** 10.1098/rspb.2015.0964

**Published:** 2015-07-22

**Authors:** Yoshihiko Onda, Kei Hashimoto, Takuhiro Yoshida, Tetsuya Sakurai, Yuji Sawada, Masami Yokota Hirai, Kiminori Toyooka, Keiichi Mochida, Kazuo Shinozaki

**Affiliations:** 1 Cellulose Production Research Team, RIKEN Center for Sustainable Resource Science, Kanagawa, Japan; 2 Technology Platform Division, Mass Spectrometry and Microscopy Unit, RIKEN Center for Sustainable Resource Science, Kanagawa, Japan; 3 Integrated Genome Informatics Research Unit, RIKEN Center for Sustainable Resource Science, Kanagawa, Japan; 4 Metabolic Systems Research Team, RIKEN Center for Sustainable Resource Science, Kanagawa, Japan; 5 Gene Discovery Research Group, RIKEN Center for Sustainable Resource Science, Kanagawa, Japan; 6 Biomass Research Platform Team, RIKEN Center for Sustainable Resource Science, Kanagawa, Japan; 7 Kihara Institute for Biological Research, Yokohama City University, Kanagawa, Japan

**Keywords:** *Brachypodium distachyon*, growth staging, BBCH scale, Zadoks scale, Pooideae, metabolome

## Abstract

*Brachypodium distachyon* is an emerging model plant for studying biological phenomena in temperate grasses. Study of the growth scale is essential to analyse spatio-temporal changes in molecular factors throughout the life cycle. For sensitive and robust staging based on morphology in *B. distachyon*, we demonstrated the utility of the BBCH (Biologische Bundesanstalt, Bundessortenamt and CHemical industry) scale, which is comparable to the Zadoks scale conventionally used for Triticeae crops. We compared the chronological progression of *B. distachyon* accessions Bd21 and Bd3-1, in addition to the progression of Chinese Spring wheat. The comparison of growth stages illustrates the morphological similarities and differences in the timing of life cycle events. Furthermore, we compared metabolite accumulation patterns across different growth stages and across different stress conditions using a widely targeted metabolome analysis. Metabolic profiling determined commonalities and specificities in chemical properties that were dependent on organisms, growth stages and/or stress conditions. Most metabolites accumulated equivalently in *B. distachyon* and wheat. This qualitative similarity indicated the superiority of *B. distachyon* as a model for Triticeae crops. The growth scale of *B. distachyon* should provide a conceptual framework for comparative analysis and for knowledge integration between this model grass and crops in the Pooideae subfamily.

## 1. Introduction

To compare biological observations in an organism, it is essential to define the organism's growth scale throughout its life cycle. Among developmental stages, life cycle staging has facilitated (i) the identification of phenotypic changes in mutants and/or accessions, and (ii) the investigation of spatio-temporal dynamics in quantitative changes of cellular molecules. Several studies and resources describe the developmental growth stages of model organisms such as the mouse (EMAP eMouse Atlas Project; http://www.emouseatlas.org/emap/home.html), fly [1] (Atlas of Drosophila Development; http://www.sdbonline.org/sites/fly/atlas/00atlas.htm), worm [2] (WormAtlas; http://www.wormatlas.org/hermaphrodite/introduction/Introframeset.html), rice [3] and *Arabidopsis* [4]. Life cycle staging is important for model organisms because it enables integration of data collected by various research communities.

*Brachypodium distachyon*, which belongs to the Pooideae subfamily, was proposed as a model organism for the study of biological systems in temperate grasses, dedicated biofuel crops and cool-season cereals such as wheat, barley, rye and oats [5–7]*. Brachypodium distachyon* has tractable features such as small size, simple growth requirements, self-fertility, a short life cycle and a small diploid genome size. As the whole genome sequence of *B. distachyon* is available (e.g. reference accession Bd21 [8]), several projects aimed at developing genomic resources have been initiated at various institutions [9,10]. Initial studies on grain development, drought tolerance and cell wall synthesis support the eligibility of *B. distachyon* as a focused model system for temperate grasses [11–16]. However, a major challenge with its use is determination of the gene functions required for crop production (e.g. grain yield, grain quality, stress tolerance, cell wall composition, cell wall biosynthesis and plant–pathogen interactions). To maximize the benefits of using *B. distachyon* as a model organism for temperate grasses, a sensitive and robust growth scale based on morphology is necessary for accurate determination of gene functions.

Various methodologies have been proposed to define growth stages in cereals based on external or internal morphology [17,18]. The Zadoks scale is a highly descriptive growth scale that is widely used for studies on Triticeae crops [19] and is based on definitions of the external morphology of cereal plants. It has been reported that the BBCH (**B**iologische **B**undesanstalt, Bundessortenamt and **CH**emical industry) scale [3], which is largely based upon and highly compatible with the Zadoks scale, can be used to identify and interpret phenotypic differences derived from genetic and environmental effects in *B. distachyon* [20]. In the BBCH scale, the same code is assigned to developmentally similar morphological stages for different cereal crops such as wheat, barley, oat and rye, thereby enabling the construction of a relevant framework to compare traits and gene functions across Triticeae crops. Application of the BBCH scale for defining a series of growth stages of *B. distachyon* should promote gene discovery, comparative analysis with other Triticeae crops and knowledge integration of different research projects.

Here, we demonstrate the utility of the BBCH scale for *B. distachyon*. We used the BBCH scale to compare the growth stages between *B. distachyon* accessions Bd21 and Bd3-1, in addition to Chinese Spring wheat. Furthermore, to demonstrate the benefits of growth stage comparison using the BBCH scale, we conducted a widely targeted metabolome analysis and compared accumulation patterns of metabolites across different growth stages and under different stress conditions in the *B. distachyon* accessions and the wheat cultivar Chinese Spring.

## 2. Material and methods

### (a) Plant material and growth conditions

Dry seeds of diploid accessions of *B. distachyon* Bd21 and Bd3-1 were provided by the National Plant Germplasm System of USDA-ARS. The dry seeds were sown on damp filter paper in a plastic Petri dish, incubated at 4°C in the dark for 3 days to synchronize germination, and then germinated in a growth chamber (MLR-350HT, Sanyo, Osaka, Japan) at 25°C under a day length of 16 h at approximately 100 µmol m^−2^ s^−1^. The germinated shoots were transplanted to pots filled with autoclaved Pro-Mix Mycorryzae (Premier Tech, Quebec, Canada). Plants were grown under controlled conditions of 22°C with a day length of 20 h at approximately 100 µmol m^−2^ s^−1^. Relative humidity was maintained at 40–60%. Plants were irrigated and fertilized every 3–4 days with 5000-fold-diluted Professional HYPONeX 10-30-20 (Hyponex Japan, Osaka, Japan). The developmental stages observed during the life cycle of *B. distachyon* (electronic supplementary material, table S1 and figure S1) were adapted to the BBCH decimal coding system based on morphology according to a previous report [20].

### (b) Statistical analysis

Statistical differences in the days from imbibition to reach each BBCH growth stage were analysed between accessions of *B. distachyon* by *t*-test (*p* < 0.05). These analyses were performed using R v. 2.15.0 statistical software [21].

### (c) Wiki-based information resource

To establish web-accessible information resources for imaging of each *B. distachyon* stage, we developed a wiki-based information resource ‘image library’ powered by MediaWiki, which is an open-source wiki software package written in PHP (http://www.mediawiki.org/wiki/MediaWiki). Our image library is available at the following URL: http://brachypedia.bmep.riken.jp/wiki/index.php/Image_library.

### (d) Light microscopy and scanning electron microscopy

For light microscopy, tissues were observed on a stereomicroscope (SZX7, Olympus, Tokyo, Japan), and images were obtained using a digital camera (DP21, Olympus).

For scanning electron microscopy (SEM), pollens were fixed overnight in 4% paraformaldehyde and 2% glutaraldehyde in 0.1 M cacodylate buffer (pH 7.2) and 0.15 M sucrose. After washing thrice with 0.1 M cacodylate buffer (pH 7.2), the pollens were post-fixed in 1% osmium tetroxide in 0.05 M cacodylate buffer (pH 7.2). After washing briefly with the same buffer, samples were dehydrated in a series of ethanol concentrations (12.5, 25, 50, 75 and 90%; each step for 30 min at 4°C), and stored in 100% ethanol overnight at 4°C. The ethanol was replaced with isopentyl acetate, and the samples were dried with a critical-point dryer (EM CPD030, Leica, Vienna, Austria) and coated with platinum using an ion sputter (JFC-1600, JEOL, Tokyo, Japan). The samples were then observed by SEM (SU1510, Hitachi High-Technologies, Tokyo, Japan) at 5 kV.

### (e) Metabolome analysis

*Brachypodium distachyon* (Bd21 and Bd3-1) and wheat (Chinese Spring) were grown at the same time under a day length of 20 h as described above. Plants were grown in plastic Petri dishes until the BBCH11 stage, and then transplanted to the pots and grown until BBCH15 as described above. For temperature stress treatments, plants at BBCH15 were subjected to 2, 12, 32 or 42°C for 24 h under a day length of 20 h. For salt stress treatments, plants at BBCH15 were subjected to 24 h of 100 or 500 mM NaCl treatment by bottom watering under a day length of 20 h. Seeds or the first leaves were collected from six to 15 individuals, lyophilized, and disrupted by TissueLyser (Qiagen, Tokyo, Japan). Quantitative data for metabolite accumulation were obtained by UPLC-TQS (Waters, Tokyo, Japan) as described previously [22]. Missing values of these data, which appeared when a metabolite was not detected in a sample, were replaced with the number 20 because the average noise level of our in-house LC-MS-based metabolome system is approximately 20. We performed three biologically independent measurements and used the average values of the replication. Peaks of metabolites that showed a signal-to-noise (*S*/*N*) ratio (*S*, average value of three biologically independent replicates; *N*, average value of three buffer-only injections) of more than 10 in at least one sample were selected. The resulting data matrix was subjected to hierarchical clustering analysis following transformation to binary logarithm (log_2_) values. A Venn diagram of the metabolites that showed *S*/*N* ratios of more than 10 was also generated using the same metabolic profile data. These analyses were performed using R v. 2.15.0 statistical software [21] with the ‘heatmap2’ and ‘venn.diagram’ functions, respectively.

## 3. Results and discussion

### (a) Morphology mapping between *Brachypodium distachyon* and wheat

To phenotypically analyse and integrate the growth stages of the new model *B. distachyon* into that of cereal crops, we employed the BBCH scale [3,20], which is highly compatible with the Zadoks scale [19]. The BBCH scale uses a numeric decimal code, which is divided into principal and secondary growth stages, and each decimal code indicates a growth stage [3]. These growth stages cover the development of *B. distachyon* from seed imbibition through the completion of flowering and seed maturation [20]. Electronic supplementary material, table S1, lists the BBCH codes and their descriptions investigated in this study. The days from seed imbibition to reach each stage when *B. distachyon* accession Bd21 and Bd3-1 were grown under controlled environmental conditions (22°C, 20 h day length, 100 µmol m^−2^ s^−1^) are also shown in electronic supplementary material, table S1. To make the *B. distachyon* growth scale and morphological properties at each stage publicly accessible, we also established an image library based on the BBCH growth scale that archives images as a wiki-based information resource (electronic supplementary material, figure S2; http://brachypedia.bmep.riken.jp/wiki/index.php/Image_library). The wiki system enables the development of web-accessible information resources that are useful in openly integrating global human knowledge. According to discussions on growth stages and further observations based on various growth conditions in the research community, the definition of the growth scale may be improved or fine-tuned. The flexible hyperlink function of the wiki system, which could provide links between the consolidated growth scale and any spatio-temporal datasets from various biological measurements, should facilitate the establishment of a heuristic resource for this species.

### (b) Comparison of life cycle between *Brachypodium distachyon* and wheat

**Figure 1.**
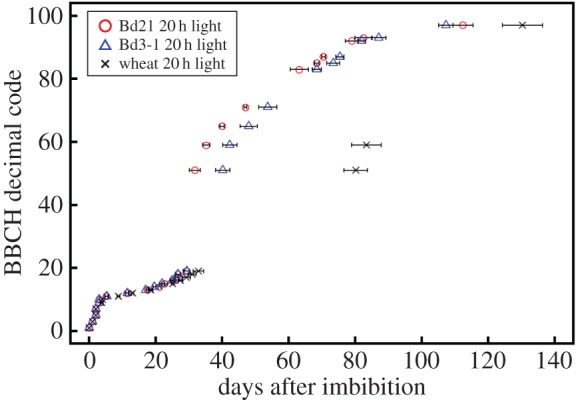
Chronological growth stages of the BBCH scale in Bd21, Bd3-1 and Chinese Spring wheat. Days between imbibition and each BBCH growth stage are indicated by horizontal bars (±s.d.). Red, blue and black markers represent Bd21, Bd3-1 and wheat, respectively, under a day length of 20 h.

Using our observation data for plant growth under a day length of 20 h, the number of days from seed imbibition to reach each growth stage was compared between Bd21 and Bd3-1 (electronic supplementary material, table S1). Differences in the days required to reach each growth stage were statistically evaluated by *t*-test (*p* < 0.05; electronic supplementary material, table S1). To compare the velocity of each stage between the *B. distachyon* accessions and wheat cultivar Chinese Spring, we measured some representative points of the BBCH scale (figure 1). At the early growth stage from BBCH00 to BBCH12, there was no significant difference in growth rate between Bd21 and Bd3-1 (figure 1; electronic supplementary material, table S1). At the middle of tillering stage (BBCH23–BBCH26), statistically significant differences were observed between Bd21 and Bd3-1 (electronic supplementary material, table S1). Regarding the tillering of *B. distachyon*, we observed differences in the number of tillers depending on accessions (six tillers in Bd21 and more than nine tillers in Bd3-1). These observations suggest that tillering of *B. distachyon* could be a phenotypic difference associated with genetic variation between these two accessions, and serve as a typical example of the usage of a growth scale to identify phenotypic differences, which are efficiently detectable throughout the life cycle. Next, we observed inflorescence emergence and heading stages. Bd21 reached BBCH51 and 59 stages significantly earlier than Bd3-1. These observations are consistent with a previous report by Schwartz *et al.* [23], who also reported that Bd21 and Bd3-1 were in the same genotypic class based on a sequence analysis of single nucleotide polymorphisms in BdVRN2 intron 1, and flowering time in both accessions was not influenced by vernalization treatment [23]. Furthermore, *B. distachyon* accessions Bd21 and Bd3-1 reached the BBCH51 and BBCH59 stages in half of the time required for Chinese Spring wheat, or less (figure 1). After inflorescence emergence (BBCH51), although the difference between Bd21 and Bd3-1 gradually decreased towards the BBCH97 stage (figure 1), all of the observed stages from BBCH51 to BBCH97 were evaluated as showing significant differences between Bd21 and Bd3-1 (electronic supplementary material, table S1). At the senescence stage, although Bd3-1 plants were 5 days behind in ripening stage (BBCH83–BBCH87), Bd3-1 plants reached the BBCH97 stage 5 days earlier than Bd21 plants. Because the BBCH97 stage is defined as plant death and collapsing, the observed results may not be due to a periodic difference during seed ripening, but rather may be attributed to a periodic difference in senescence associated with the genotype.

### (c) Flowering and seed development

**Figure 2.**
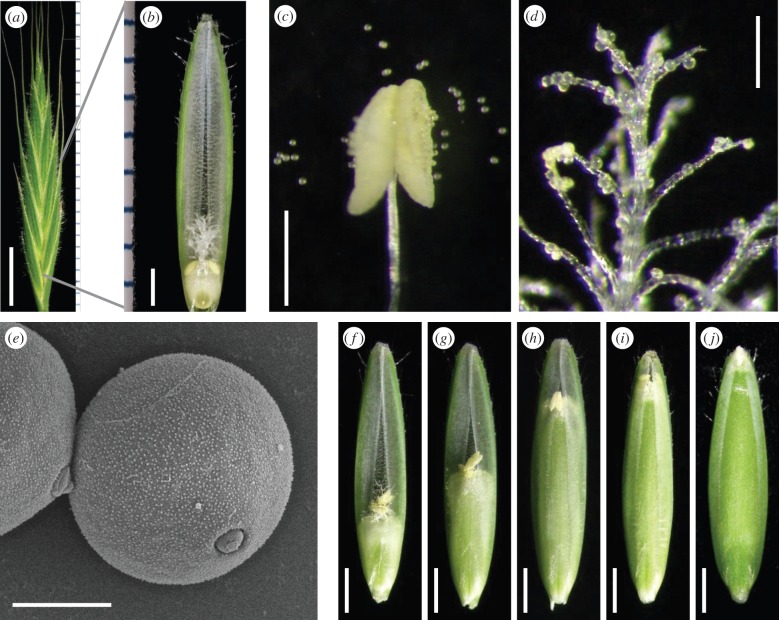
Flower structure and endosperm development. (*a*) Spikelet. (*b*) Floret. (*c*) Dehiscent anther and pollen grains. (*d*) Pollen grains attached to the stigma. (*e*) SEM image of the pollen surface structure. (*f–j*) Endosperm development towards BBCH71 (*i*) and BBCH73 (*j*). Scale bars: (*a*) 5 mm; (*b*) 1 mm; (*c*) 500 µm; (*d*) 200 µm; (*e*) 10 µm; (*f*–*j*) 1 mm.

The growth stages of flowering were effectively defined by the codes of the BBCH scale (figure 2). A spikelet of *B. distachyon* constitutes two glumes and several florets. The floret comprises two bracts (a lemma on the outside and a palea on the inside), which enclose a double-plumed stigma and only two anthers (figure 2*a*,*b*). Generally, grass species (including the genus *Brachypodium*, except *B. distachyon*) contain three anthers in a floret [24]; this is one of the most notable morphological differences in florets between *B. distachyon* and the cereal crops in Poaceae. At the BBCH69 stage, the anther dehisced and shed a number of pollen grains (figure 2*c*). Some of the pollen grains attached to the stigma and fertilization proceeded (figure 2*d*). The spherical pollen grain had a pebbly texture and an aperture on the pollen wall (figure 2*e*). After fertilization, the endosperm of self-compatible *B. distachyon* [25] gradually developed along the growth stages towards BBCH73 (figure 2*f*–*j*). At the BBCH71 stage, although the floret was almost completely filled with endosperm (figure 2*i*), the endosperm was still thinner and softer (electronic supplementary material, figure S3A) than in the later stages (electronic supplementary material, figure S3B–H). The endosperm remarkably expanded until BBCH75 (electronic supplementary material, figure S3B,C). At BBCH77, the endosperm contained more solids in the milk (electronic supplementary material, figure S3D). At BBCH83, dough development had started (electronic supplementary material, figure S3E). Grain drying and filling gradually proceeded (electronic supplementary material, figure S3F,G,H).

### (d) Comparative metabolome analysis in *Brachypodium distachyon* and wheat

**Figure 3.**
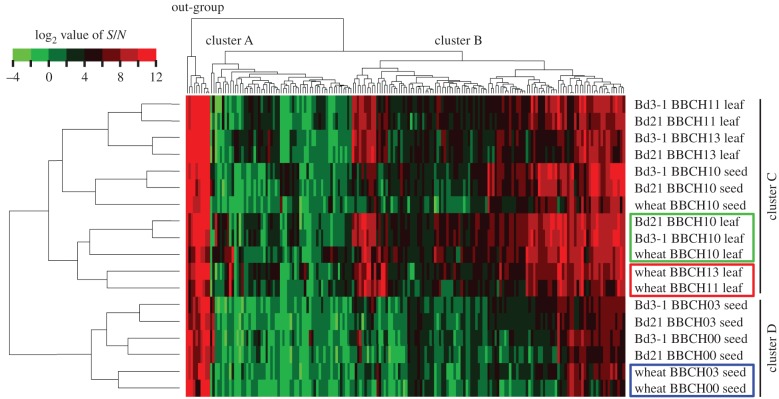
Metabolite profiling of the seeds and leaves of Bd21, Bd3-1 and Chinese Spring wheat at different growth stages. Dendrogram and heat map show metabolite accumulation, clustered based on the binary logarithm (log_2_) values of quantitative data, which were obtained by UPLC-TQS. Only the first leaves were used as leaf samples.

In addition to the morphological definition, accumulation patterns of chemical compounds could be molecular markers that describe cellular condition with metabolic signatures [26]. Integration of metabolomic properties and morphological description of plant developmental stages provides a crucial molecular context to compare cellular systems among related plant species, which could facilitate crop molecular breeding. According to our observations, the growth rates of earlier stages were not markedly different among *B. distachyon* accessions and wheat (figure 1). Therefore, we performed a widely targeted metabolome analysis focused only on earlier growth stages (BBCH00 seed, BBCH03 seed, BBCH10 seed, BBCH10 leaf, BBCH11 leaf and BBCH13 leaf), and compared metabolite accumulation patterns among *B. distachyon* accessions and Chinese Spring wheat. To obtain comprehensive metabolome profiles, we applied a platform for widely targeted metabolome analysis developed at RIKEN [22]. In total, 517 metabolites were detected in the seed and/or leaf samples (electronic supplementary material, table S2). Metabolites that showed *S*/*N* ratios of more than 10 in at least one sample among 18 (three plant types, six growth stages) were selected (indicated with asterisks in electronic supplementary material, table S2), and a data matrix containing 182 metabolites was created (electronic supplementary material, table S3) for comparison of metabolite accumulation patterns. Hierarchical clustering analysis revealed two major clusters and an out-group (figure 3, cluster A, B and out-group). Cluster A contained metabolites with relatively lower accumulation among all subjected samples, whereas cluster B contained metabolites with relatively higher accumulation. The out-group contained metabolites with markedly higher accumulation among all subjected samples. The hierarchical clustering analysis also grouped samples separately into seed and leaf, with the exception of the BBCH10 seed (figure 3, cluster C and cluster D). Because the first leaf emerged through the coleoptile at the BBCH10, the clustering results suggest that the BBCH10 seed was already losing its metabolic identity as a seed. Apart from this exception, the results suggest that our metabolome and hierarchical clustering analyses were accurate in efficiently elucidating the differences in species, growth stages and tissues such as seed and leaf. For metabolite accumulation in seeds, Bd21 and Bd3-1 were grouped under the same cluster at the BBCH00 and BBCH03 stages; however, wheat formed a unique cluster (figure 3, enclosed by a blue line). This result suggests that the difference in seed metabolite characteristics between *B. distachyon* and wheat may reflect a difference in metabolic systems in the seeds. For metabolite accumulation in leaves, although Bd21 and Bd3-1 were always grouped in the same clusters at the same growth stages, *B. distachyon* and wheat were grouped in the same cluster only at the BBCH10 stage (figure 3, enclosed by a green line). At the BBCH11 and BBCH13 stages, wheat formed a unique cluster (figure 3, enclosed by a red line). These results suggest that metabolite accumulation in younger leaves reflects common characteristics of Pooideae or Poaceae, whereas metabolite accumulation in older leaves is affected by differences in species. To summarize the commonalities and specificities of metabolome profiling, metabolites that showed an *S*/*N* ratio of more than 10 were visualized in a Venn diagram at each stage (electronic supplementary material, figure S4). The majority of the metabolites accumulated among Bd21, Bd3-1 and wheat at all growth stages; these commonly accumulated metabolites are listed in electronic supplementary material, table S4.

**Figure 4.**
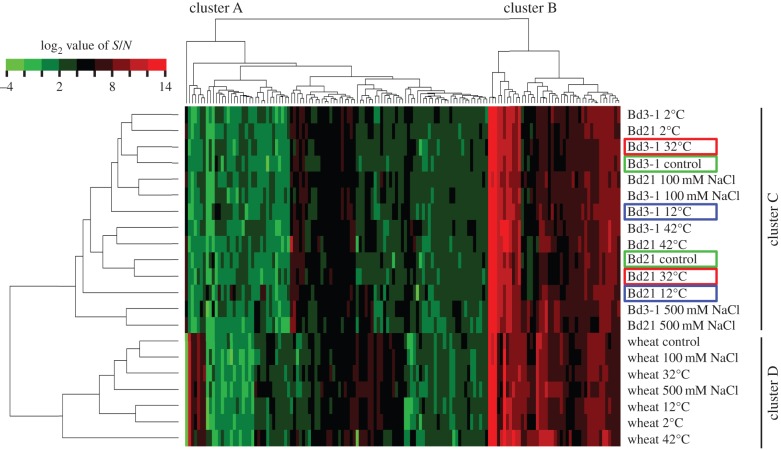
Metabolite profiling of the leaves of Bd21, Bd3-1 and Chinese Spring wheat under stress conditions. Dendrogram and heat map show metabolite accumulation, clustered based on the binary logarithm (log_2_) value of quantitative data obtained by UPLC-TQS. Only the first leaves were used.

Next, we conducted further metabolome analyses under stress conditions (2°C, 12°C, 32°C, 42°C, 100 mM NaCl and 500 mM NaCl) using plants at the BBCH15 stage. In total, 491 metabolites were detected in the leaf samples (electronic supplementary material, table S5). Metabolites that showed *S*/*N* ratios of more than 10 in at least one sample among 21 (three plant types, seven stress conditions) were selected (indicated with asterisks in electronic supplementary material, table S5), and a data matrix containing 145 metabolites was created (electronic supplementary material, table S6). Hierarchical clustering analysis revealed two major clusters (figure 4, cluster A and B). Cluster A contained metabolites with relatively lower accumulation among all subjected samples, whereas cluster B contained metabolites with relatively higher accumulation. The hierarchical clustering analysis also grouped samples separately into *B. distachyon* and wheat (figure 4, cluster C and D). This separation of these two species at BBCH15 is consistent with the results of the hierarchical clustering analysis at BBCH13 as described above. Although samples of Bd21 subjected to the 2°C, 42°C, 100 mM NaCl and 500 mM NaCl treatments were grouped with those of Bd3-1 in the same cluster, Bd21 samples subjected to control, 12 and 32°C treatments were grouped separately from those of Bd3-1 (figure 4, enclosed by green, blue and red lines, respectively). These results suggest that Bd21 and Bd3-1 showed similar metabolite accumulation profiles as *B. distachyon* under more stringent stress conditions, whereas they showed different profiles as different accessions under milder stress conditions. To summarize the commonalities and specificities of metabolome profiling, metabolites that showed an *S*/*N* ratio of more than 10 were visualized in a Venn diagram under each stress condition (electronic supplementary material, figure S5). The majority of the metabolites accumulated among Bd21, Bd3-1 and wheat under all treatments; these commonly accumulated metabolites are listed in electronic supplementary material, table S7.

In our metabolome analysis, the majority of metabolites accumulated among Bd21, Bd3-1 and wheat even at different growth stages and under different growth conditions. This qualitative similarity of accumulated metabolites indicates the superiority of *B. distachyon* as a model for Triticeae crops for breeding improved crop varieties. Although there were no remarkable differences between Bd21 and Bd3-1 because they grouped into the same clusters at the same growth stages (figures 3 and 4), differences in the quantity of metabolite accumulation were observed between these accessions (electronic supplementary material, figures S4 and S5). Furthermore, we carefully analysed the difference in metabolite accumulation between Bd21 and Bd3-1 by using the same data matrix used for hierarchical clustering analysis. Betaine, sucrose and melibiose were three of the most differently accumulated metabolites in both the dry seed (BBCH00) and first leaf (BBCH11, 13 and 15) (electronic supplementary material, table S8). These differences in metabolite accumulation between *B. distachyon* accessions could represent molecular phenotypes that could be genetically analysed by quantitative trait locus analysis or genome-wide association studies to identify causal genes and polymorphisms associated with metabolite accumulation.

### (e) Utility of the BBCH scale

Life cycle staging serves as a developmental landmark and a trigger for the collection of experimental materials that are of interest at specific developmental stages, even among independent research projects. A sensitive and robust method for life cycle staging is essential; thus, the BBCH scale provides a consolidated developmental context that would enable growth stages in *B. distachyon* to be distinguished (electronic supplementary material, table S1 and figure S2). This growth scale can also be easily adapted to other genetic populations such as mutants, natural variants and mapping populations. Furthermore, it is known that a defined growth scale is also useful to evaluate growth under particular environmental conditions including abiotic and biotic stress in addition to nutritional and phytohormonal stimuli. Herein, we demonstrated the utility of the BBCH scale by comparing metabolite accumulation profiles under different stress conditions between Bd21 and Bd3-1, which suggests the usefulness of the growth scale for omics analyses among different mutants or accessions in a consolidated developmental context. Recently, sodium azide-induced mutants, the TILLING platform [27] (http://www-urgv.versailles.inra.fr/tilling/brachypodium.htm) and T-DNA insertion lines [28] have been available for *B. distachyon*. Furthermore, different types of accession can be used among diverse natural genetic variations [29,30]. With the combined use of the consolidated growth scale and these developed resources, *B. distachyon* could be used to accelerate gene discovery research and comprehensive characterization of gene function, leading to crop improvement in grasses.

The BBCH scale was developed for crops such as wheat, barley, oat and rye. This consistency across key cereals is useful when comparing developmental stage-dependent morphology or orthologous gene expression between *B. distachyon* and other Pooideae plants. Although the current version of the BBCH scale is defined only by morphological observations, integration of observations from various spectra should improve the compatibility of the growth scale between *B. distachyon* and its targeted crops. Recently, accessibility of whole-genome sequences in Triticeae plants has been considerably improved. The whole genomes of barley, common wheat and diploid ancestors of wheat were deciphered in succession from 2012 to 2014 [9,31–38]. These updates of Triticeae genomics should promote the discovery of genes involved in productivity in these corps. There is also an increased need for *B. distachyon* as a model plant for rapid functional analyses of discovered genes. Comparative views of developmental stage-dependent morphology and various omics analyses between *B. distachyon* and wheat and barley should enable not only further elucidation of biological systems, particularly in the Pooideae subfamily, but also immediate utilization of the knowledge from the model grass to improve the productivity of these staple grains.

## Supplementary Material

Table S1.xls

## Supplementary Material

Table S2.xls

## Supplementary Material

Table S3.xls

## Supplementary Material

Table S4.xls

## Supplementary Material

Table S5.xls

## Supplementary Material

Table S6.xls

## Supplementary Material

Table S7.xls

## Supplementary Material

Table S8.xls

## Supplementary Material

Fig. S1_Final.pdf

## Supplementary Material

Fig. S2_Final.pdf

## Supplementary Material

Fig. S3_Final.pdf

## Supplementary Material

Fig. S4_Final.pdf

## Supplementary Material

Fig. S5_Final.pdf

## Data accessibility

Data on metabolome analysis are available at Data Resources of Plant Metabolomics (DROP Met) accessible via the Platform for RIKEN Metabolomics (PRIMe) website at http://prime.psc.riken.jp.
