# Supplementary figures and images for: Determination of growth stages and metabolic profiles in Brachypodium distachyon for comparison of developmental context with Triticeae crops
Source: Proc Biol Sci. 2015 Jul 22;282(1811):20150964. doi: 10.1098/rspb.2015.0964 (PMC4528556; doi:10.1098/rspb.2015.0964)

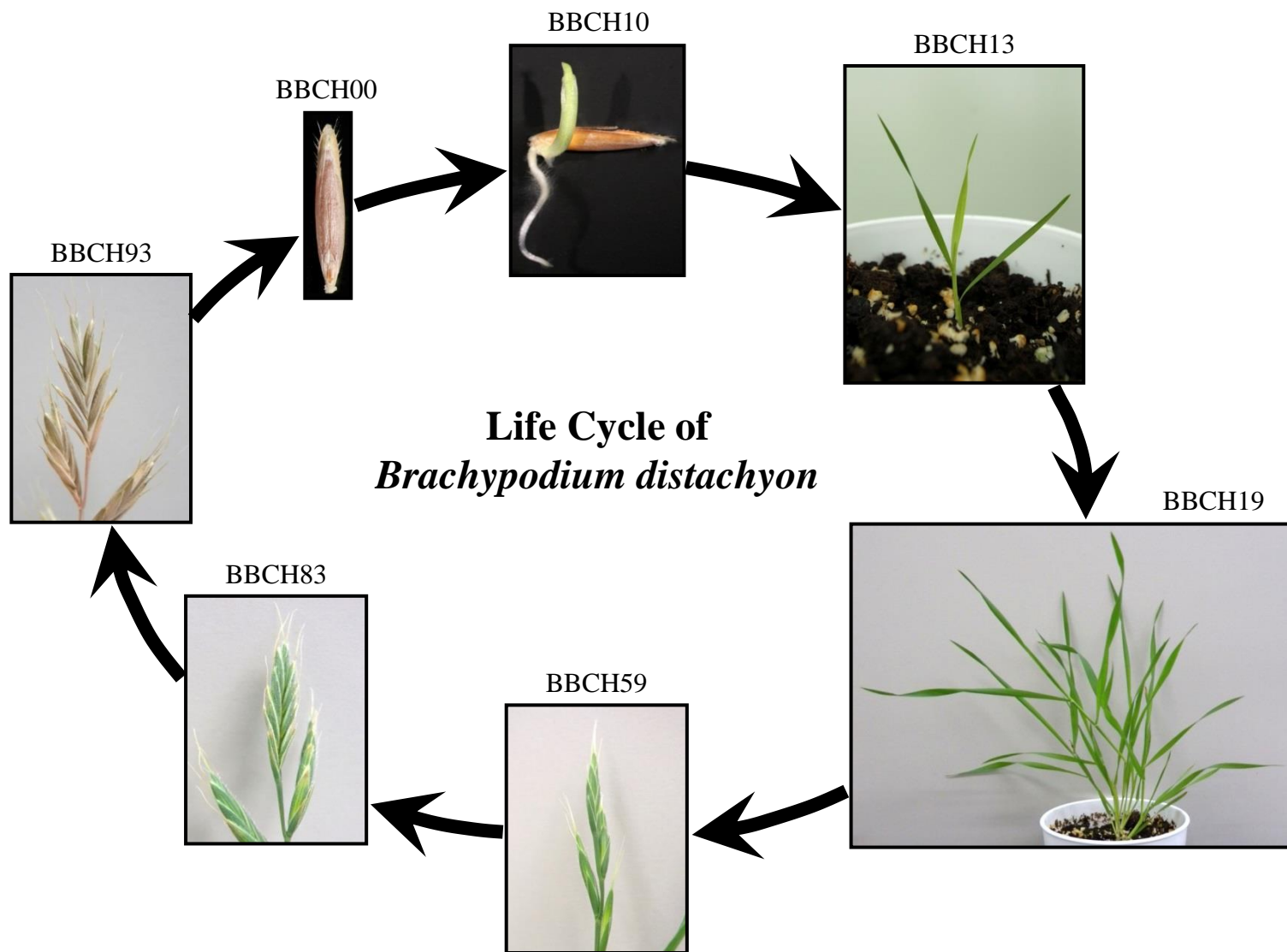

Fig. S1

Supplement: Fig. S1_Final.pdf [file rspb20150964supp9.pdf]

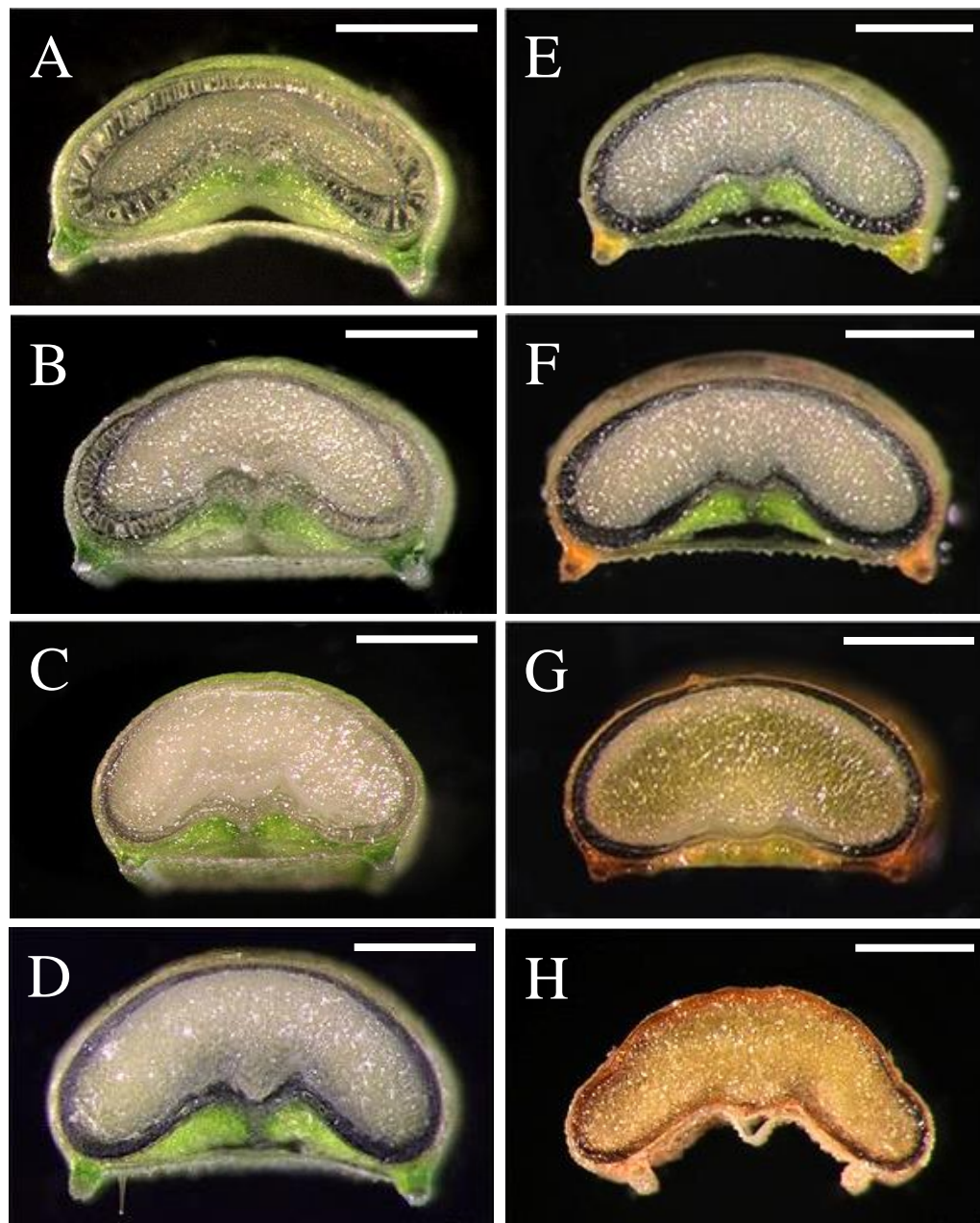

Fig. S3

Supplement: Fig. S3_Final.pdf [file rspb20150964supp11.pdf]

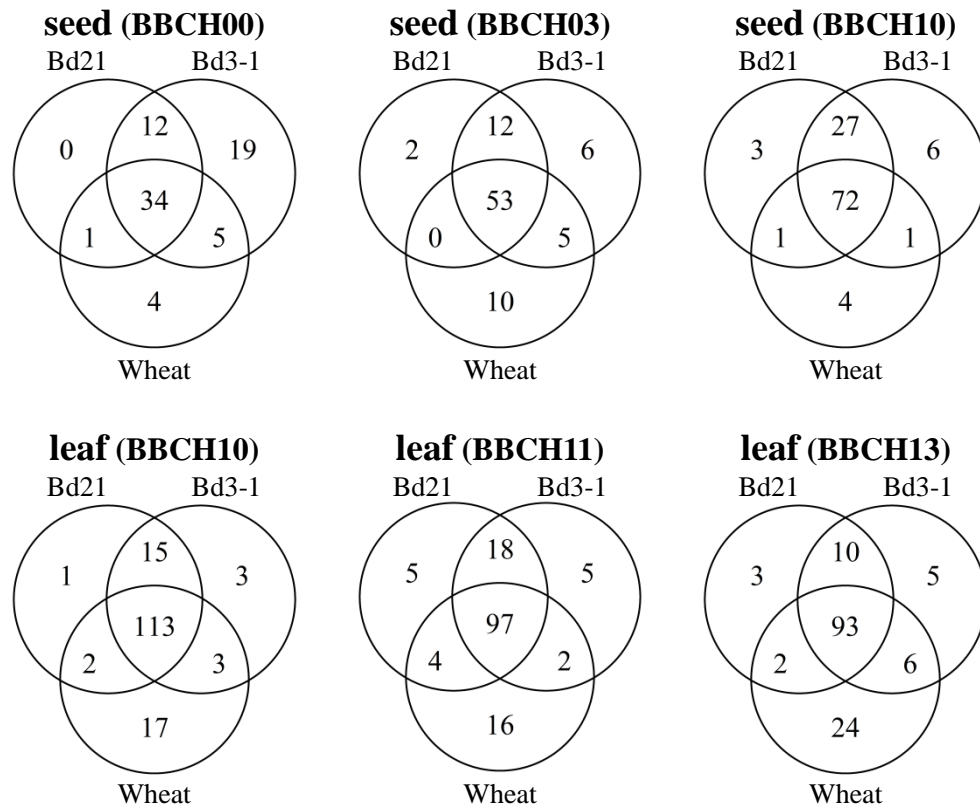

Fig. S4

Supplement: Fig. S4_Final.pdf [file rspb20150964supp12.pdf]

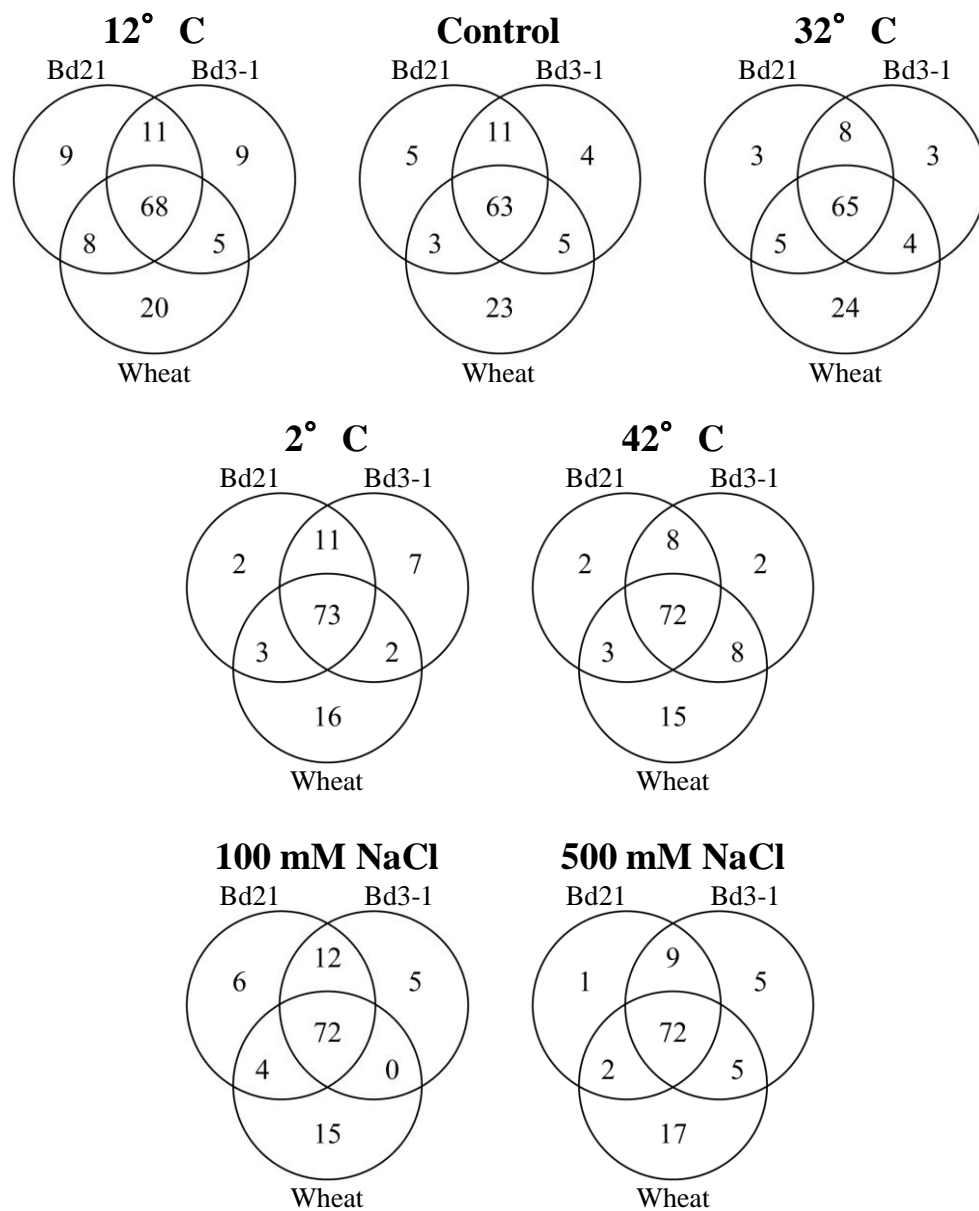

Fig. S5

Supplement: Fig. S5_Final.pdf [file rspb20150964supp13.pdf]
