# Supplementary material for: Determination of growth stages and metabolic profiles in Brachypodium distachyon for comparison of developmental context with Triticeae crops
Source: Proc Biol Sci. 2015 Jul 22;282(1811):20150964. doi: 10.1098/rspb.2015.0964 (PMC4528556; doi:10.1098/rspb.2015.0964)
Supplement: Fig. S2_Final.pdf [file rspb20150964supp10.pdf]

Stage images

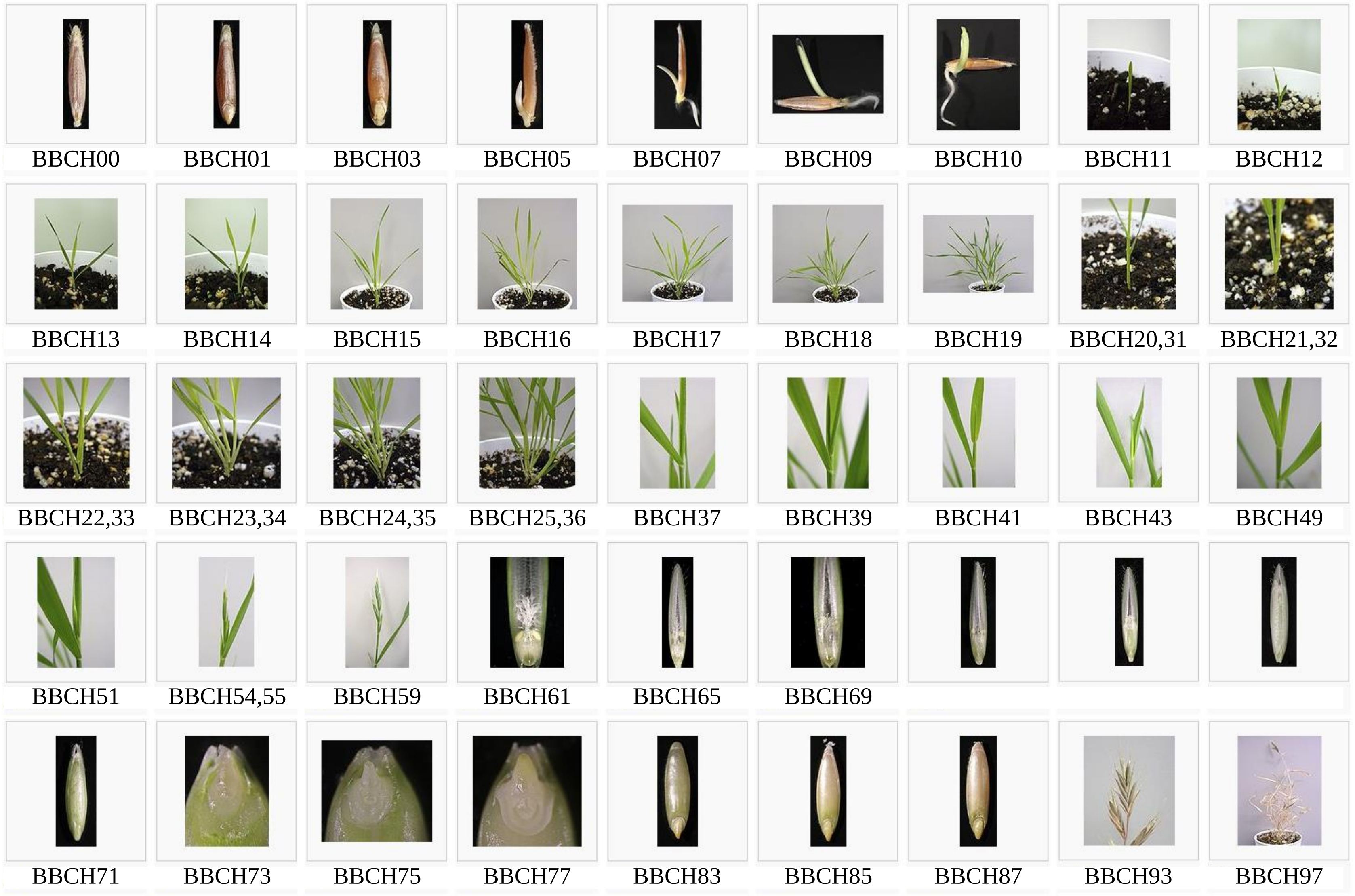

http://brachypedia.bmep.riken.jp/wiki/index.php/Image_library

Fig. S2
